# Effects of low versus standard pressure pneumoperitoneum on renal syndecan-1 shedding and VEGF receptor-2 expression in living-donor nephrectomy: a randomized controlled study

**DOI:** 10.1186/s12871-020-0956-7

**Published:** 2020-02-04

**Authors:** Dita Aditianingsih, Chaidir Arif Mochtar, Aida Lydia, Nuryati Chairani Siregar, Nur Ita Margyaningsih, Amir Sjarifuddin Madjid, Suhendro Suwarto

**Affiliations:** 1 Department of Anesthesiology and Intensive Care, Cipto Mangunkusumo Hospital, Universitas Indonesia, Jakarta, Indonesia; 2 Department of Anesthesiology, Cipto Mangunkusumo Hospital, Salemba Raya 6th, Jakarta, 10430 Indonesia; 3 Department of Urology, Cipto Mangunkusumo Hospital, Universitas Indonesia, Jakarta, Indonesia; 4 Department of Internal Medicine, Division of Nephrology and Hypertension, Cipto Mangunkusumo Hospital, Universitas Indonesia, Jakarta, Indonesia; 5 Department of Anatomical Pathology, Cipto Mangunkusumo Hospital, Universitas Indonesia, Jakarta, Indonesia; 6 Eijkman Institute of Molecular Biology, Jakarta, Indonesia; 7 Department of Internal Medicine, Division of Tropical and Infectious Disease, Cipto Mangunkusumo Hospital, Universitas Indonesia, Jakarta, Indonesia

**Keywords:** Pneumoperitoneum, Renal resistive index, Interleukin-6, Syndecan-1, sVEGFR-2, Laparoscopic nephrectomy

## Abstract

**Background:**

Laparoscopic nephrectomy is a preferred technique for living kidney donation. However, positive-pressure pneumoperitoneum may have an unfavorable effect on the remaining kidney and other distant organs due to inflamed vascular endothelium and renal tubular cell injury in response to increased systemic inflammation. Early detection of vascular endothelial and renal tubular response is needed to prevent further kidney injury due to increased intraabdominal pressure induced by pneumoperitoneum. Transperitoneal laparoscopic living donor nephrectomy represented a human model of mild increasing intraabdominal pressure. This study aimed to assess the effect of increased intraabdominal pressure on vascular endothelium and renal tubular cells by comparing the effects of low and standard pressure pneumoperitoneum on vascular endothelial growth factor receptor-2 (VEGFR-2) expression and the shedding of syndecan-1 as the early markers to a systemic inflammation.

**Methods:**

We conducted a prospective randomized study on 44 patients undergoing laparoscopic donor nephrectomy. Subjects were assigned to standard (12 mmHg) or low pressure (8 mmHg) groups. Baseline, intraoperative, and postoperative plasma interleukin-6, syndecan-1, and sVEGFR-2 were quantified by ELISA. Syndecan-1 and VEGFR-2 expression were assessed immunohistochemically in renal cortex tissue. Renal tubule and peritubular capillary ultrastructures were examined using electron microscopy. Perioperative hemodynamic changes, end-tidal CO_2_, serum creatinine, blood urea nitrogen, and urinary KIM-1 were recorded.

**Results:**

The low pressure group showed lower intra- and postoperative heart rate, intraoperative plasma IL-6, sVEGFR-2 levels and plasma syndecan-1 than standard pressure group. Proximal tubule syndecan-1 expression was higher in the low pressure group. Proximal-distal tubules and peritubular capillary endothelium VEGFR-2 expression were lower in low pressure group. The low pressure group showed renal tubule and peritubular capillary ultrastructure with intact cell membranes, clear cell boundaries, and intact brush borders, while standard pressure group showed swollen nuclei, tenuous cell membrane, distant boundaries, vacuolizations, and detached brush borders.

**Conclusion:**

The low pressure pneumoperitoneum attenuated the inflammatory response and resulted in reduction of syndecan-1 shedding and VEGFR-2 expression as the renal tubular and vascular endothelial proinflammatory markers to injury due to a systemic inflammation in laparoscopic nephrectomy.

**Trial registration:**

ClinicalTrials.gov NCT:03219398, prospectively registered on July 17th, 2017.

## Background

Minimally invasive surgery is increasingly performed in many institutions. The increased intra-abdominal pressure (IAP) that occurs as a result of pneumoperitoneum insufflation may have an unfavorable effect on the kidney and other distant organs. Laparoscopic nephrectomy is a less-invasive technique for living donor allograft kidney procurement and has become a preference to promote early postoperative recovery [1, 2]. As these laparoscopic techniques advance, more living donors are undergoing surgery to save others. As such, the postoperative condition of these donors becomes a priority. It is important to ensure safety and minimize surgical risk in both the kidney recipient and donor [3].

The mean IAP in a healthy patient while supine is 1.8 mmHg with a range between − 1 to 6 mmHg [4]. The World Society of the Abdominal Compartment Syndrome (WSACS) defines the upper normal limit for IAP to be approximately 5–7 mmHg in adults [5]. The kidneys are at risk of injury induced by increased IAP secondary to pneumoperitoneum-induced renal venous congestion and compression of the renal vasculature and parenchyma [6]. A prospective clinical study on living transperitoneal laparoscopic donor nephrectomy with 12 mmHg IAP showed an increased inflammatory response and early signs of kidney injury when compared with open retroperitoneal nephrectomy patients [3]. Additionally, an animal study applying pneumoperitoneum to isolated perfused rat kidneys demonstrated early onset inflammation and renal apoptosis [7]. The decreased renal blood flow leads to tissue hypoperfusion that triggers an inflammatory response. After desufflation, reperfusion occurs when renal blood flow is normalized. This further stimulates the synthesis of inflammatory cytokines, which have been postulated to mediate the association between blood flow changes and endothelial and epithelial cell injury [6]. Vascular endothelial dysfunction and tubular cell injury in response to inflammatory cytokines play an important role in acute kidney injury (AKI) [7].

Syndecan-1 is a cell surface proteoglycan that consists of a heparan and chondroitin sulphate which is expressed on various epithelial and vascular endothelial cells. Syndecan-1 is involved in many cellular functions that promote cell proliferation and survival, and its shedding may be an important proponent in the mechanism that is responsible for tubular epithelial injury in ischemic and inflammatory conditions. Elevated serum syndecan-1 has predicted AKI and mortality in patients with acute heart failure and in cardiac surgery [8, 9]. Higher tubular epithelial syndecan-1 expression promotes tubular cell survival and repair, that is correlated with prolonged allograft survival in kidney transplant patients [10]. Activation of vascular endothelial growth factor (VEGF) binding to VEGF receptor-2 (VEGFR-2) has an important role in maintaining angiogenesis and microvasculature permeability [9]. Overstimulation of VEGF-VEGFR-2 induces renal tubulointerstitial injury through altered endothelial proliferation, abnormal angiogenesis, and extracellular matrix deposition [10]. These findings indicate that the inhibition of syndecan-1 shedding and VEGF-VEGFR-2 stimulation are novel targets in preventing or managing AKI, since serum blood urea nitrogen (BUN), creatinine, and urine output are delayed signs of deteriorating kidney function [8].

We hypothesized that short-term increases in intraabdominal pressure could alter renal perfusion and induce a systemic inflammatory response that leads to tubular cell injury. In the current investigation, we aimed to evaluate the effect of low pressure pneumoperitoneum on vascular endothelium and renal tubular cells markers induced by a systemic inflammatory response during transperitoneal laparoscopic living donor nephrectomy. We further hypothesized that using a lower pressure pneumoperitoneum could reduce these effects. Here, we compared the effects of low and standard pressure pneumoperitoneum on shedding of syndecan-1 and activated vascular endothelial growth factor receptor-2 (VEGFR-2) expression, as the early vascular endothelial and renal tubular proinflammatory markers in response to the presence of systemic inflammatory cytokines. The primary outcome was detecting the plasma level and tubular expression of syndecan-1. The secondary outcomes were VEGFR-2 and soluble VEGFR-2 (sVEGFR-2) expression in renal tubuloendothelial cells, plasma interleukin-6 (IL-6), and urinary KIM-1 content.

## Methods

### Ethical considerations

A prospective single-blind clinical study on patients undergoing transperitoneal laparoscopic living donor nephrectomy was conducted at the university teaching hospital after receiving approval from the medical ethics committee (protocol no. 17-06-0619, approval date: June 19th, 2017). This study was registered prospectively on July 17th, 2017 in ClinicalTrials.gov (NCT:03219398)..

### Patient enrollment

We enrolled 44 patients between August 2017 and February 2018. All patients provided written informed consent prior to participation. The inclusion criteria were age between 18 and 65 years, American Society of Anesthesiologist (ASA) physical status classification I–II, and a body mass index (BMI) of 18–25 kg/m^2^. Exclusion criteria were hemodynamic instability defined as the changes of mean arterial pressure or cardiac index > 25% below or above baseline despite intervention treatment, significant bleeding causing failure to maintain pressure, and conversion of laparoscopy to open nephrectomy. Patients were allocated using blocked randomization (https://www.sealedenvelope.com/simple-randomiser/v1/lists) with a block size of 4. Then, using a list of random numbers in sealed envelopes, patients were divided into 12 mmHg (standard pressure) or 8 mmHg (low pressure) pneumoperitoneum groups. Both the patients and principal investigator were blinded to group allocation. The principal investigator received the randomization codes after all measurements and calculations of all patients had been entered into the results database.

### Anesthesia and Pneumoperitoneum

All patients were continuously monitored by bedside telemetry of heart rate, non-invasive blood pressure, pulse oxygen saturation, end-tidal carbon dioxide (IntelliVue MP70 Philips Healthcare, Netherlands), and cardiac output relates to body surface area (BSA) using bioimpedance cardiometry (ICON™, Osypka Germany). After midazolam premedication, standardized anesthesia was induced with 1–2 mg/kg intravenous propofol and 1 μg/kg intravenous fentanyl. Intubation was facilitated with 0.5 mg/kg intravenous atracurium. General anesthesia maintenance was performed using sevoflurane with an end-tidal sevoflurane target of 1.5–2% (Aisys C2, GE Healthcare, Illinois, USA) to maintain a bispectral index value between 40 and 50 (BIS™, Covidien, Minneapolis, USA). Maintenance with 0.005 mg/kg^/^min intravenous atracurium and 2 μg/kg/hour fentanyl was conducted to achieve train of four between 0.15 and 0.25 (TOF-Watch, Organon, Ireland).

All patients received bilateral ultrasound-guided transmuscular quadratus lumborum block that was performed by two anesthetist consultants (See Additional file 1). Under general anesthesia, the patient was positioned in the lateral decubitus position. The research assistant then opened the sealed envelope and allocated the patient into the standard or low pressure group based on inclusion number. After introducing the Hasson trocar, pneumoperitoneum was established by carbon dioxide (CO_2_) insufflation. The patients received 8 or 12 mmHg pneumoperitoneum pressure (Olympus Medical System Corp, UHI-4, Tokyo Japan) depending on their randomization. The surgeon inserted an endoscopic 30° video and introduced two 5-mm and 10- or 12-mm laparoscopic trocars under direct vision. Details of port placement and surgical space conditions during 8 and 12 mmHg pressure pneumoperitoneum can be viewed in Additional file 2 and Additional file 3. In this study, all patients underwent left kidney procurement. The kidney was extracted through the Pfannenstiel incision using an endobag and was immediately flushed with a cold preservative solution (Custodiol® HTK). At the end of surgery, the pneumoperitoneum was desufflated and the incision was closed. All patients received bilateral QL block using 0.25% bupivacaine before extubation. The patients received a reversal of muscle relaxant if necessary and were extubated. In this study, all anesthesia and surgery were performed by the same consultant team with comparable distributions.

### Sample collection and analysis

Intrarenal Doppler using a 3.5–5 MHz ultrasound transducer (Logic 7-GE, USA) was used to measure interlobar arterial peak systolic and end diastolic velocities, and the resistive index (RI) was calculated by peak systolic velocity minus end diastolic velocity and divided by peak systolic velocity. RI measurements were performed on the left kidney before anesthesia induction (baseline), intraoperatively at 2 h of pneumoperitoneum, and on the remaining right kidney 2 h after gas desufflation.

Brachial vein venous blood samples and urine samples were collected at the same time of RI measurements. All samples were stored at -80^o^ C until analysis, and each sample was run in duplicate. Plasma IL-6, syndecan-1, and sVEGFR-2 were analyzed by ELISA (Human IL-6, Quantikine®, R&D, Minneapolis USA, Human CD138/Syndecan-1, Diaclone, France, and Human VEGF R2/KDR Quantikine® R&D) following manufacturer’s instructions. KIM-1 was determined from a 10 μL urine specimen and was measured by ELISA (Human Urinary KIM-1, Quantikine®, R&D). Perioperative hemodynamic profiles represented by heart rate, systolic pressure, diastolic pressure, mean arterial pressure, and cardiac output were recorded at the same times as blood sample collection. Pre-postoperative serum creatinine and BUN were also recorded.

### Immunohistochemistry and renal ultrastructure examination

Cold ischemic time was defined as the interval between kidney immersion in ice and intravascular perfusion with cold preservative solution. One renal biopsy was performed at the end of this cold ischemic time. Tissues were immersed in Dubosq solution for 30 min and fixed in 10% neutral-buffered formalin, embedded in paraffin, and sectioned. For syndecan-1 immunostaining, 4 μm sections were stained using periodic acid Schiff. Sections were incubated with Anti-Syndecan-1 primary antibody (B-A38, ab714, Abcam, USA) overnight at 4 °C. For VEGFR-2 immunostaining, sections were incubated with Anti-VEGFR-2/KDR primary antibody (SP123, ab115805, Abcam) overnight at 4 °C. After washing, sections were incubated with horseradish peroxidase conjugated secondary antibody for 30 min at room temperature. The slides were then washed and incubated with 3,3-Diaminobenzidine (DAB)-peroxidase substrate solution for 20 s.

Protein expression of syndecan-1 and VEGFR-2 was determined by immunohistochemistry, observed under a light microscope (Leica DM500) and photographed with a digital camera (Leica ICC50 HD, Germany). On each slide, 20 different fields (× 400 magnification) were selected. The semiquantitative analysis of syndecan-1 expressions in the proximal and distal tubular epithelial cells was performed using HER-2 score and H-Score. Five hundred proximal and distal tubular cells were assessed on each slide. Tagging and evaluation of intensity (0–3+) of these 500 cells were based on HER-2 criteria (0: no staining; 1+: weak and incomplete membrane staining in less than 10% of the cells; 2+: weak complete staining of the membrane in more than 10% of the cells; 3+: strong complete homogenous membrane staining in more than 30% of the cells) with the help of the ImageJ software. This scoring was converted into percentages and entered into the histological score (H-score) formula; H-score = [3 x strong intensity cell percentage (3+)] + [2 x medium intensity cell percentage (2+) + [1 x weak intensity cell percentage (1+]. The resulting value equates to between 0 and 300 [11]. VEGFR-2 expression in arterial endothelial cells, peritubular and glomerular capillaries, podocyte cells, and proximal and distal tubular epithelial cells was assessed. Semiquantitative analysis was performed by scoring the percentage of positive VEGFR-2 expression in 25 peritubular arteries and 50 peritubular capillaries in each sample. VEGFR-2 expression in proximal and distal tubular epithelial cells was assessed using HER-2 score and H-Score, as described above. All scoring was performed by three observers who were blinded to sample randomization.

Electron microscopy (EM) was performed to examine the ultrastructure of proximal tubules, distal tubules, peritubular capillaries, and arteries. After perfusion fixation with 4% paraformaldehyde, kidney tissue was fixed in 2.5% glutaraldehyde and postfixed with 2% osmium tetroxide in 2.5% K_3_Fe(CN)_6_ and 3% sucrose. The samples were dehydrated in graded ethanol, embedded in Spurr resin, and vacuumed. Ultrathin sections were stained with 2% uranyl acetate with triple lead citrate and examined by EM (JEOL 1010, Tokyo, Japan) at 80 kV.

### Statistical analysis

Sample calculations were performed based on a preliminary study containing 5 patients in each group (total of 10 patients) assessing effects of reductions in plasma syndecan-1 and sVEGFR-2 levels and previous study [12]. Power analysis (α = 0.05, β = 0.20) with a 20% reduction in plasma syndecan-1 (SD ± 47) and sVEGFR-2 (SD ± 2062.32) was used to determine the sample size of 20 patients per group. A total sample size of 44 subjects was considered sufficient to allow for a 10% dropout.

A Chi-squared test was used for categorical variables. Parametric data were presented as the mean ± standard deviation or median (interquartile range) and were compared using unpaired t-test or Mann-Whitney test. Repeated analysis of variance followed by post hoc analysis was also performed. Transformed data were analyzed and presented as geometric means and 95% confidence interval (minimum–maximum) using a general linear model. All analysis was performed using SPSS 20.0 software. *P*-value < 0.05 was considered statistically significant.

## Results

**Fig. 1:**
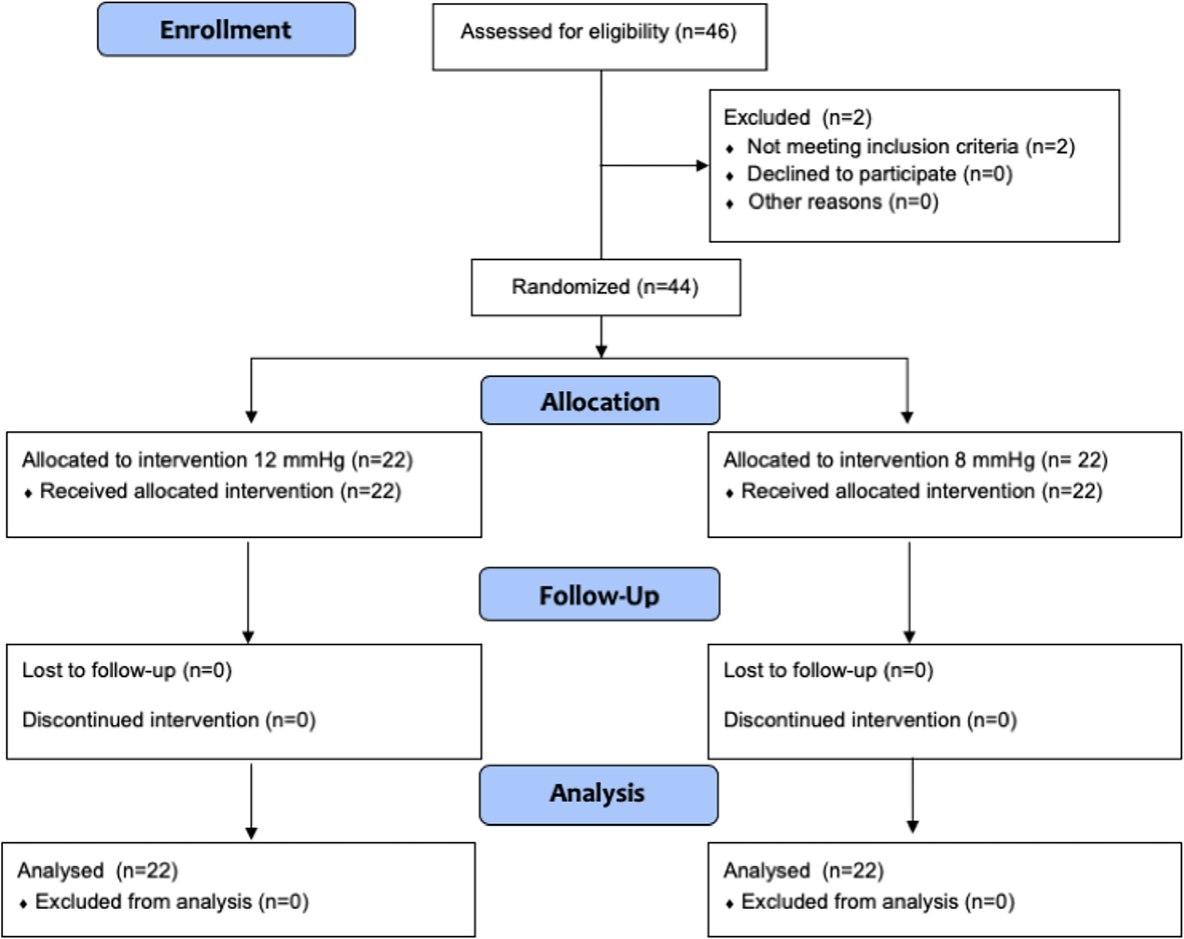
CONSORT flow diagram

Patients were recruited between August 2017 and February 2018. The CONSORT flow diagram is presented in Fig. 1. After exclusion of 2 patients, 44 patients were enrolled and analyzed.

All baseline and perioperative characteristics are presented in Table 1. Sex, age range, BMI, baseline BUN and creatinine levels were not significantly different between the two groups.

**Table 1:** Patients characteristics data

Characteristics	12 mmHg group (*n* = 22)	8 mmHg group (*n* = 22)	*p*-Value
Sex			
Male (n, %)	10 (45.5)	15 (68.2)	0.2
Female (n, %)	12 (54.5)	7 (31.8)	
Age	31.5 (30.97–39.31)	30.50 (29.52–39.93)	0.7
Weight (kg)	63.67 ± 9.02	60.19 ± 12.31	0.3
Height (cm)	159.80 ± 6.97	164.05 ± 8.27	0.1
Body Mass Index (BMI)	25.75 ± 5.37	22.23 ± 3.26	0.009
Pre-operative			
BUN (mg/dL)	20.00 (17.36–22.28)	23.50 (20.82–25.82)	0.044
Creatinine (mg/dL)	0.80 (0.72–0.96)	0.85 (0.78–0.96)	0.4

Categorical variables are presented in n (%). Numerical variables are presented as mean (± standard deviation) or median and confidence interval 95% (minimum–maximum), *p* < 0.05 is significant. The two groups were compared with Chi-Square Test or unpaired t-test or Mann-Whitney U test

Table 2 shows that hemodynamic cardiac index (CI), stroke volume index (SVI), mean arterial pressure (MAP), and end-tidal CO_2_ were not significantly different between the 12 mmHg and 8 mmHg groups. However, heart rate (HR) in the 12 mmHg group was significantly higher than that in the 8 mmHg group during insufflation pneumoperitoneum and after desufflation.

**Table 2:** Intraoperative hemodynamic parameters and *end-tidal* CO_2_

Parameters	Mean (CI 95%)		*p*	Mean Difference CI 95%	*p*
	12 mmHg	8 mmHg			
1. Cardiac index (L/minute/m^2^)					
a. baseline	2.84 (2.49–3.23)	2.76 (2.48–3.07)	0.7	1.028 (0.872–1.211)	
b. at 2 h of pneumoperitoneum	3.20 (2.78–3.70)	3.35 (2.98–3.77)	0.6	0.957 (0.800–1.146)	0.6
c. 2 h after desufflation	3.24 (2.85–3.69)	3.39 (3.08–3.73)	0.6	0.957 (0.818–1.119)	
2. Stroke volume index (mL/m^2^)					
a. baseline	36.84 (32.77–41.67)	34.70 (31.05–38.47)	0.5	0.690 (0.240–1.980)	
b. at 2 h of pneumoperitoneum	33.70 (28.98–38.550	37.20 (32.34–42.06)	0.1	0.33 (0.10–1.03)	0.5
c. 2 h after desufflation	32.12 (28.57–35.77)	37.64 (32.6–42.77)	0.1	0.35 (0.11–1.10)	
3. End-tidal CO_2_ (mmHg)					
a. baseline	35.59 (33.89–37.29)	34.86 (33.47–36.25)	0.5	0.727 (−1.41–2.86)	
b. at 2 h of pneumoperitoneum	37.77 (36.36–39.18)	38.00 (36.79–39.21)	0.8	− 0.23 (− 2.03–1.57)	0.3
c. 2 h after desufflation	37,14 (35.46–38.81)	38.32 (37.06–39.58)	0.3	− 1.182 (− 3.22–0.85)	
4. Mean arterial pressure (mmHg)					
a. baseline	77.92 (72.75–83.10)	78.30 (72.85–83.76)	0.9	−0.38 (−7.68–6.92)	
b. at 2 h of pneumoperitoneum	80.05 (75.72–84.37)	82.31 (77.59–87.15)	0.5	−2.26 (− 8.47–3.95)	0.5
c. 2 h after desufflation	85.77 (80.48–91.07)	83.06 (78.79–87.31)	0.4	2.73 (−3.87–9.32)	
5. Heart rate (beats/minute)					
a. baseline	76.82 (72.34–81.56)	65.69 (61.56–70.10)	0.1	1.17 (0.07–1.27)	
b. at 2 h of pneumoperitoneum	86.98 (82.62–91.56)	74.10 (69.41–79.10)	< 0.001	1.18 (1.08–1.27)	0.033
c. 2 h after desufflation	95.39 (89.62–101.53)	77.66 (72.23–83.52)	< 0.001	1.23 (1.12–1.35)	

Data are presented as percentage (%) or geometric mean and confidence interval 95% (minimum–maximum), *p* < 0.05 is significant. The two groups were compared with unpaired t-test and a general linear model

Table 3 shows the non-statistically significant difference in the duration of pneumoperitoneum, surgery, anesthesia, warm and cold ischemic time, and postoperative urine output between the two groups. The 12 mmHg group showed intraoperative pressure stability in 14 patients and pressure loss during suctioning or instrumentation in 8 patients that needed intermittent pneumoperitoneum pressure increase, which were significantly different than the 8 mmHg group that showed the pressure stability in 3 patients and pressure loss in 19 patients. None of the subject analyzed had a significant bleeding during the procedure that needed pneumoperitoneum pressure increase.

**Table 3:** Patients intraoperative and postoperative data

Parameter	12 mmHg group (*n* = 22)	8 mmHg group (*n* = 22)	*p*-Value
Intraoperative:			
Duration of pneumoperitoneum (minute)	260 (242.78–285.22)	254 (238.49–270.51)	0.5
Duration of surgery (minute)	290 (272.78–315.22)	282 (263.35–300.98)	0.4
Duration of anesthesia (minute)	288 (273.04–316.05)	300 (259.02–292.31)	0.1
First warm ischemic time (minute)	3.50 (2.50–3.40)	3.51 (2.51–3.42)	0.8
Cold ischemic time (minute)	25.40 (25.33–27.47)	25.40 (24.94–27.08)	0.7
Pneumoperitoneum pressure stability:			
Stable (n, %)	14 (63.64)	3 (13.64)	0.002
Loss of pressure (n, %)	8 (36.36)	19 (86.36)	
Post-operative:			
BUN (mg/dL)	25.50 (24.07–28.29)	26.00 (25.09–31.00)	0.3
Creatinine (mg/dL)	1.10 (1.02–1.26)	1.20 (1.05–1.32)	
Urine output (ml/kg/hour)	1.04 (0.91–1.55)	1.26 (1.11–1.43)	0.8
One year follow up:			
BUN (mg/dL)	24.99 (21.94–29.54)	29.33 (24.88–34.58)	0.2
Creatinine (mg/dL)	1.10 (0.94–1.23)	1.29 (0.87–2.29)	0.1

Categorical variable presented in n (%). Numerical variable presented with median and confidence interval 95% (minimum–maximum), *p* < 0.05 is significant. The two groups were compared with Fisher test or Mann-Whitney U test

**Fig. 2:**
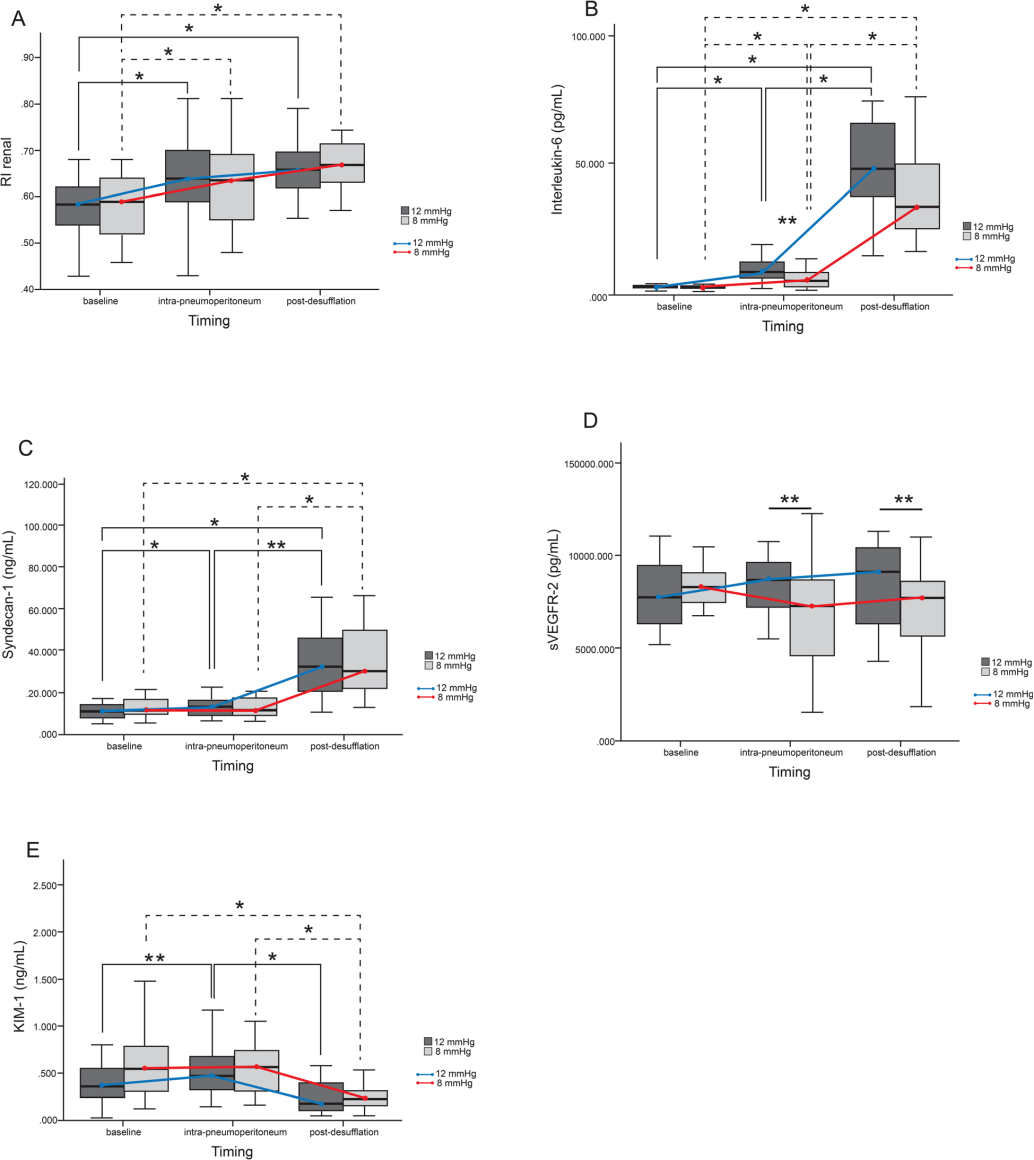
Comparison of renal resistive index (RI), plasma interleukin-6 (IL-6), syndecan-1, soluble VEGFR-2, and urinary KIM-1 between 12 mmHg and 8 mmHg groups. **a** RI. 12 mmHg vs 8 mmHg: 2-h pneumoperitoneum (0.66 (0.63–0.68) vs 0.67 (0.65–0.70), *p* = 0.4); 2 h desufflation (0.66 (0.64–0.68) vs 0.68 (0.66–0.70), *p* = 0.4). Compared to baseline (12 mmHg: 0.59 (0.55–0.62); 8 mmHg: 0.60 (0.55–0.61)) 2-h pneumoperitoneum and 2-h desufflation were significantly higher (*p* < 0.001). **b** IL-6 (pg/dL). 12 mmHg vs 8 mmHg: 2-h pneumoperitoneum (8.92 (6.21–11.62) vs 4.75 (3.50–5.99), *p* = 0.003); 2-h desufflation (46.17 (35.36–56.98) vs 37.42 (27.89–46.95), *p* = 0.2). Compared to baseline: (12 mmHg: 1.66 (1.41–1.90); 8 mmHg: 1.50 (1.31–1.69)) 2-h pneumoperitoneum and 2-h desufflation were significantly higher (*p* < 0.001). **c** Syndecan-1 (ng/mL). 12 mmHg vs 8 mmHg: 2-h pneumoperitoneum 15.18 (11.14–19.22) vs 13.66 (10.04–17.27), *p* = 0.1); 2-h desufflation (12 mmHg: 30.52 (23.80–37.23) vs 33.12 (25.21–41.02), *p* = 0.9). Compared to baseline: (12 mmHg: 10.87 (8.81–12.92); 8 mmHg: 12.07 (9.56–14.57)) 2-h pneumoperitoneum and 2-h desufflation were significantly higher (*p* < 0.001). **d** sVEGFR-2 (pg/dL). 12 mmHg vs 8 mmHg: 2-h pneumoperitoneum 8106.02 (7187.38–9024.66) vs 6841.05 (5598.85–8083.25), *p* = 0.032), 2-h desufflation (8452.25 (7486.88–9417.61) vs 7263.92 (6258.32–8269.51); *p* = 0.044) **e** KIM-1 (ng/mL). 12 mmHg vs 8 mmHg: 2-h pneumoperitoneum (0.47 (0.33–0.60) vs 0.51 (0.38–0.64), *p* = 0.7), 2-h desufflation (0.20 (0.12–0.27) vs 0.21 (0.15–0.27), *p* = 0.7). Compared to baseline: (12 mmHg: 0.32 (0.18–0.45); 8 mmHg: 0.52 (0.36–0.68)) 2-h pneumoperitoneum and 2-h desufflation were significantly different (*p* < 0.001). All data are presented as geometric mean and confidence interval 95% (minimum–maximum). Continuous data was analyzed using repeated ANOVA. Between-group comparisons were analyzed using unpaired t-test and a general linear model; * *p* < 0.001, ** *p* < 0.05

Figure 2 shows a between-group comparison of changes in renal RI values, plasma IL-6, syndecan-1, sVEGFR-2, and urinary KIM-1 at baseline, 2 h of pneumoperitoneum and 2 h after desufflation (See Additional file 4). Perioperatively, RI was not significantly different between the 12 and 8 mmHg pressure groups during 2 h of pneumoperitoneum (0.66 (0.63–0.68) vs 0.67 (0.65–0.70), *p* = 0.4); 2 h after desufflation 0.66 (0.64–0.68) vs 0.68 (0.66–0.70), *p* = 0.4). In both groups, when compared to baseline of 12 mmHg (0.59 (0.55–0.62)) and 8 mmHg (0.60 (0.55–0.61)), RI was significantly increased during 2 h of pneumoperitoneum and 2 h after desufflation (*p* < 0.001). When compared to those in the 12 mmHg group, plasma IL-6 levels in the 8 mmHg group were significantly lower during pneumoperitoneum (4.75 (3.50–5.99) vs 8.92 (6.21–11.62) pg/mL; *p* = 0.003) and 2 h after desufflation (37.42 (27.89–46.95) vs 46.17 (35.36–56.98) pg/mL; *p* = 0.2). Compared to baseline values of 12 mmHg (1.66 (1.41–1.90)) and 8 mmHg (1.50 (1.31–1.69)) pg/mL, plasma IL-6 levels were significantly increased during 2 h of pneumoperitoneum almost 4–8 times greater (*p* < 0.001), and increased 5–8 times more at 2 h after (*p* < 0.001). Plasma syndecan-1 levels were non-significantly lower in the 8 mmHg group than in the 12 mmHg group during 2 h of pneumoperitoneum (13.66 (10.04–17.27) vs 15.18 (11.14–19.22) ng/mL; *p* = 0.1) and at 2 h after desufflation (33.12 (25.21–41.02) vs 30.52 (23.80–37.23) ng/mL; *p* = 0.9). Compared to baseline value of 12 mmHg (10.87 (8.81–12.92)) and 8 mmHg (12.07 (9.56–14.57)) ng/mL, plasma syndecan-1 levels were significantly increased during 2 h of pneumoperitoneum (*p* < 0.001), and increased 2 times further 2 h after desufflation (*p* < 0.001). Plasma sVEGFR-2 was significantly lower in the 8 mmHg group compared to that in the 12 mmHg group, during 2 h of pneumoperitoneum (6841.05 (5598.85–8683.83) vs 8106.02 (7187.38–9024.66) pg/mL; *p* = 0.032) and 2 h after desufflation (7263.92 (6258.32–8269.51) vs 8452.25 (7486.88–9417.61) pg/mL; *p* = 0.044). Urinary KIM-1 level in the 8 mmHg group were not significantly different than the 12 mmHg group during 2 h of pneumoperitoneum (0.51 (0.38–0.64) vs 0.47 (0.33–0.60) ng/mL, *p* = 0.7) and 2 h after desufflation (0.21 (0.15–0.27) vs 0.20 (0.12–0.27) ng/mL, *p* = 0.7). Compared to baseline values of 12 mmHg (0.32 (0.18–0.45)) and 8 mmHg (0.52 (0.36–0.68)) ng/mL urinary KIM-1 was significantly higher during 2 h of pneumoperitoneum (*p* < 0.001) and significantly decreased 2 h after desufflation in both groups (*p* < 0.001).

**Fig. 3:**
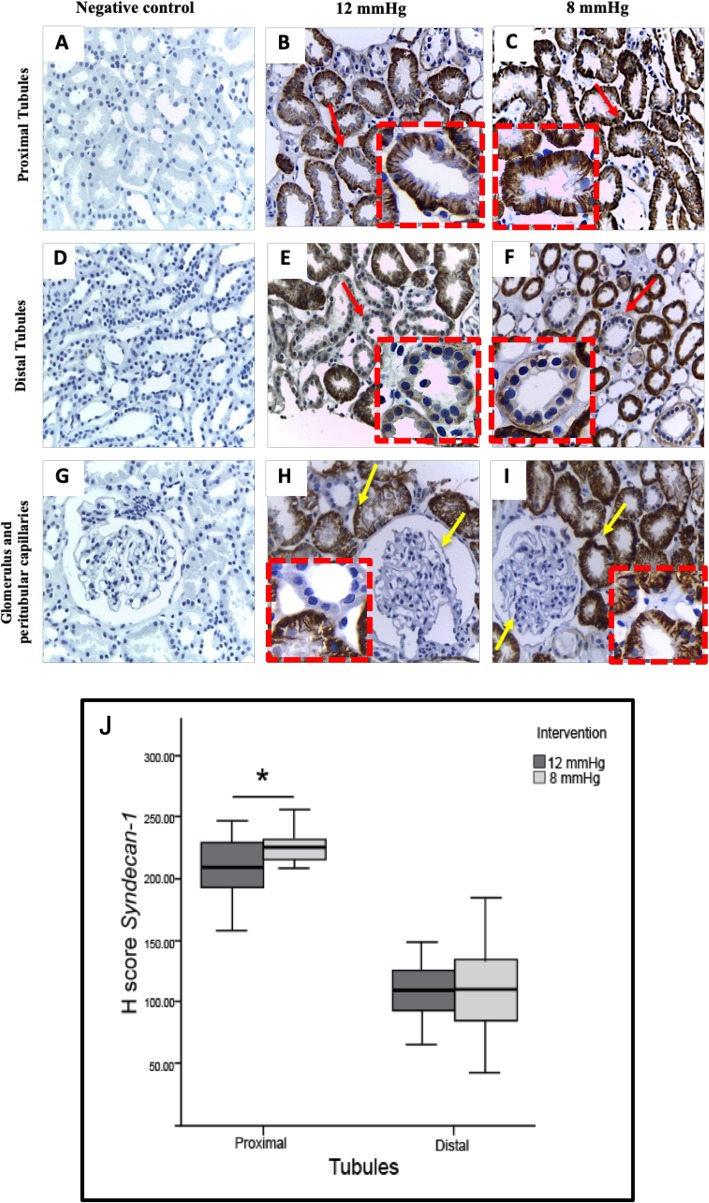
Syndecan-1 expression of tubular epithelial cells in 12 mmHg and 8 mmHg groups. **a**, **d**, **g** Negative control. **b** Reduced intensity of proximal tubule syndecan-1 expression in the 12 mmHg group. **c** Proximal tubule syndecan-1 expression is weaker in the 12 mmHg group than in the 8 mmHg group. **e** Syndecan-1 expression between the distal tubule of the 12 mmHg group and **f** the 8 mmHg group was not different. **h**, **i** Syndecan-1 expression is negative in the glomerular and peritubular capillaries of both pressure groups. Original magnification was × 400, and red dashed boxes show a higher magnification. Red arrows indicate positive syndecan-1 expression, yellow arrows indicate negative syndecan-1 expression. **j** The H-score of proximal tubule syndecan-1 expression is lower in the 12 mmHg group than the 8 mmHg group (211.00 (199.05–219.67) vs 225.90 (215.46–231.50), *p* = 0.030), and is not significantly different between groups in the distal tubules (108.10 (98.49–118.31) vs 112.80 (94.53–128.12), *p* = 0.8). Data are presented as median (95% CI). The two groups were compared using Mann-Whitney test; * *p* < 0.05

Figure 3 shows the H-score of proximal tubule syndecan-1 expression was significantly higher in the 8 mmHg group than in the 12 mmHg group (225.90 (215.46–231.50) vs 211.00 (199.05–219.67); *p* = 0.03). The H-score of syndecan-1 expression in the distal tubules was non-statistically significant different between the 8 mmHg and 12 mmHg groups (112.80 (94.53–128.12) vs 108.10 (98.49–118.31); *p* = 0.8). In both groups, syndecan-1 expression was negative in glomerular and peritubular capillaries (See Additional file 4).

**Fig. 4:**
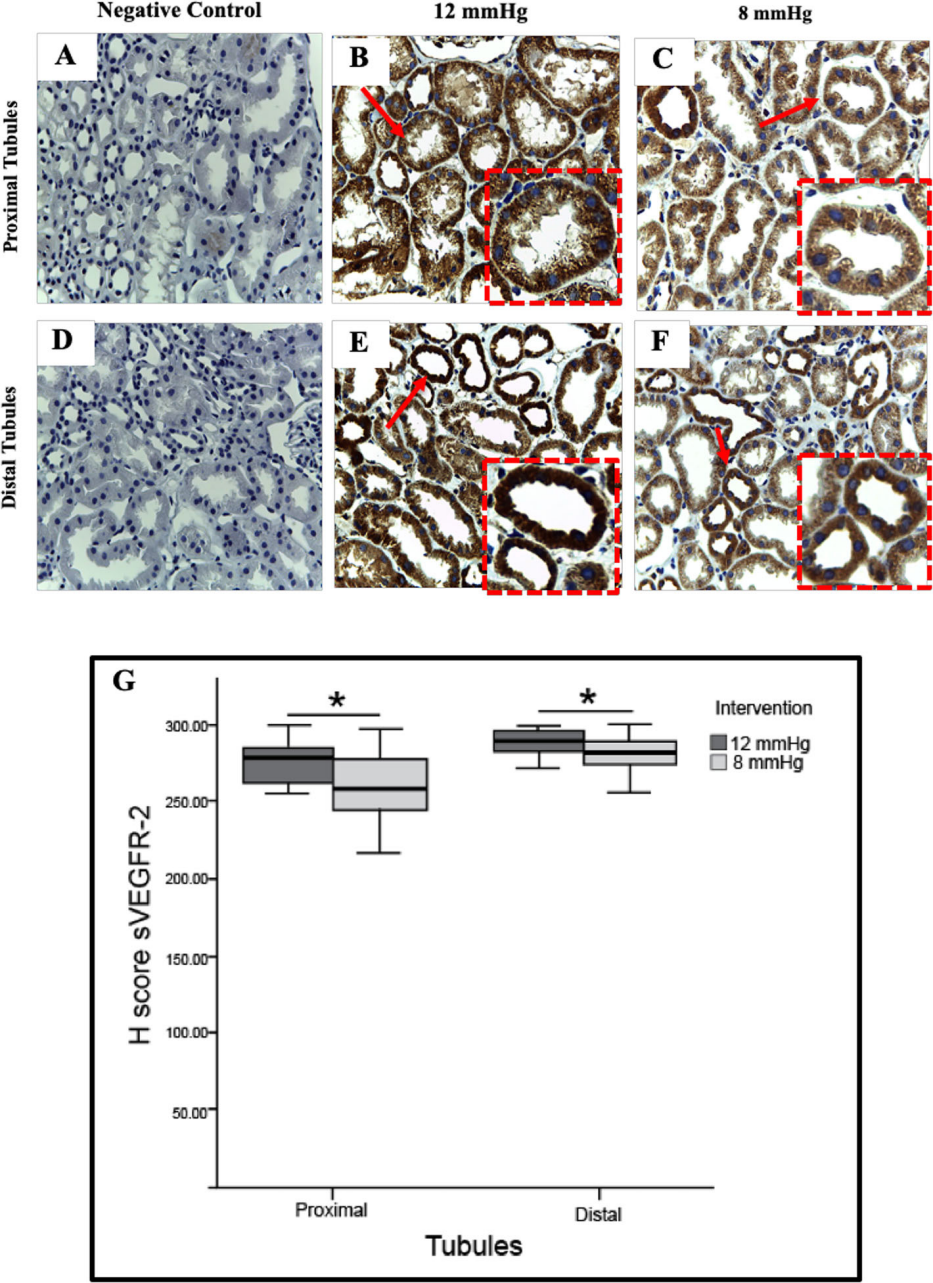
Tubular epithelial VEGFR-2 expression in the 12 mmHg and 8 mmHg groups. **a**, **d** Negative control. **b** Increased proximal tubule VEGFR-2 expression in the 12 mmHg group. **c** Proximal tubule VEGFR-2 expression is stronger in the 12 mmHg group than in the 8 mmHg group. **e** Increased distal tubule VEGFR-2 expression in the 12 mmHg group. **f** Distal tubule VEGFR-2 expression is stronger in the 12 mmHg group than in the 8 mmHg group. Original magnification was × 400, and red dashed boxes show a higher magnification. Red arrows indicate positive VEGFR-2 expression. **g** The H-score of proximal tubule VEGFR-2 expression are significantly higher in the 12 mmHg group than in the 8 mmHg group (278.00 (269.37–282.05) vs 258.80 (248.93–268.91), *p* = 0.005) and distal tubule (288.80 (282.59–291.37) vs 279.40 (271.36–284.72), *p =* 0.02). Data are presented as median (95%CI). The two groups were compared using Mann-Whitney test; * *p* < 0.05

**Fig. 5:**
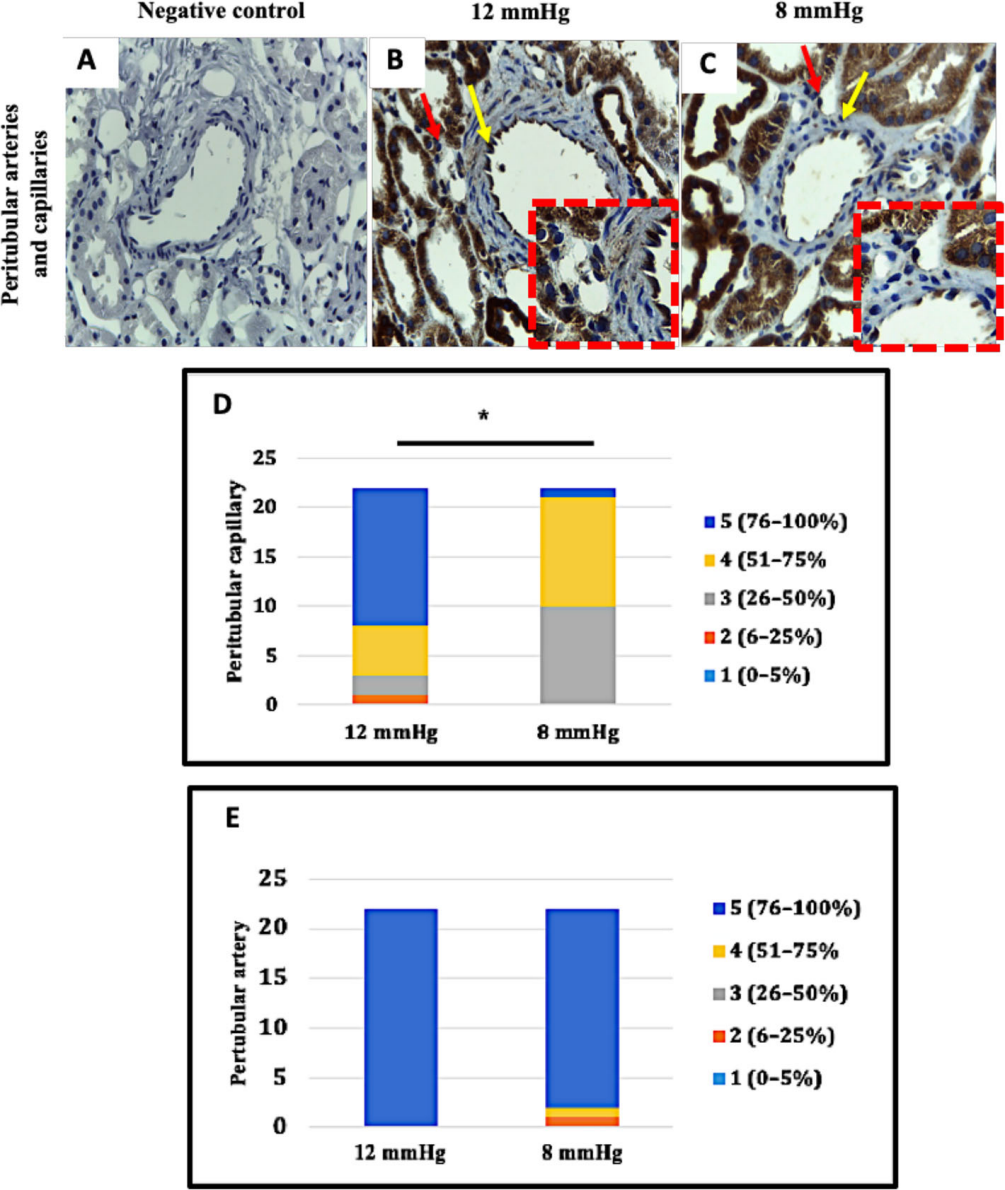
Peritubular vascular endothelial cell VEGFR-2 expression in 12 mmHg and 8 mmHg pneumoperitoneum pressure groups. **a** Negative control. **b** Strong peritubular capillary and artery VEGFR-2 expression in the 12 mmHg group. **c** Peritubular capillary and artery VEGFR-2 expression is stronger in the 12 mmHg group than in the 8 mmHg group. Original magnification was × 400, and red dashed boxes show a higher magnification. Red arrows indicate positive VEGFR-2 expression in the peritubular capillary endothelium, and yellow arrows indicate positive VEGFR-2 expression in the peritubular artery endothelium. **d** VEGFR-2 peritubular capillary expression score is significantly higher in the 12 mmHg group than 8 mmHg group (76.27 (66.53–86.02) vs 54.55 (48.56–60.53), *p* < 0.001). **e** Artery VEGFR-2 expression score is not significantly different between groups (93.27 (91.69–94.60) vs 83.27 (76.60–89.95), *p* = 0.2). Data were analyzed using Chi-square test for trends or Mann-Whitney test; * *p* < 0.001, ** *p* < 0.05

Figure 4 shows the H-score of proximal tubule VEGFR-2 expression was significantly lower in the 8 mmHg group than in the 12 mmHg group (258.80 (248.93–268.91) vs 278.00 (269.37–282.05), *p* = 0.005). The H-score of distal tubule VEGFR-2 expression was significantly lower in the 8 mmHg group than in the 12 mmHg group (279.40 (271.36–284.72) vs 288.80 (282.59–291.37), *p* = 0.024), respectively (See Additional file 4). Figure 5 shows peritubular capillary VEGFR-2 histological score comparisons showed a significantly lower percentage of strong expression cells (54.55 (48.56–60.53) vs 76.27 (66.53–86.02), *p* < 0.001) and a lower histological score in the in 8 mmHg group than in the 12 mmHg group (*p* < 0.001). Peritubular arterial endothelial cell VEGFR-2 expression was not significantly different between groups (93.27 (91.69–94.60) vs 83.27 (76.60–89.95); *p* = 0.2).

**Fig. 6:**
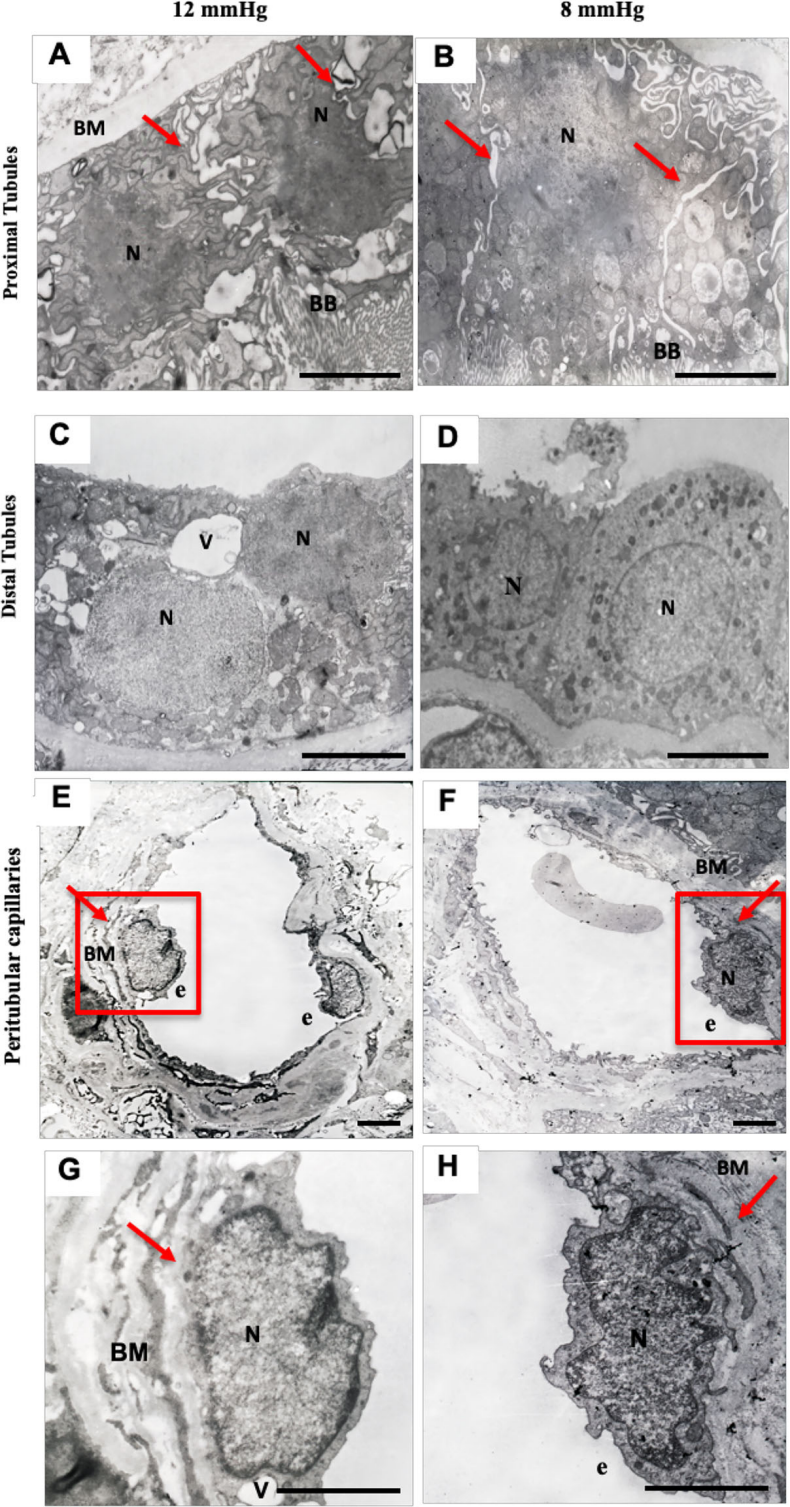
Renal tubule and peritubular capillary ultrastructure in the 12 mmHg and 8 mmHg pneumoperitoneum pressure groups. **a** Proximal tubular epithelial cells in the 12 mmHg group. Arrows show tenuous epithelial membranes and detached brush borders. **b** Proximal tubular epithelial cells in the 8 mmHg group. Arrows indicate a tight epithelial membrane and intact brush border. **c** Distal tubular epithelial cells in the 12 mmHg group show vacuolizations and a diffuse nuclear border. **d** Distal tubular epithelial cells in the 8 mmHg group show an intact nucleus and no vacuolization. **e** The peritubular capillary in the 12 mmHg group shows a swollen nucleus and edematous endothelial layer. The arrow shows a disrupted basement membrane. **f** The peritubular capillary endothelium in the 8 mmHg group shows an intact nucleus and endothelial layer. The arrow shows an intact basement membrane. The red box represents the details of images (**e**) and (**f**), and can be seen at a larger scale in (**g**) and (**h**); scale bar = 2 μm. N = nucleus, P = Podocyte, FP = Foot podocyte, BB = Brush Border, V = vacuole, BM = basement membrane, e = endothelium

Electron microscopy studies were performed to determine the early changes in tubular epithelial cells, peritubular capillaries, and glomerulus ultrastructure. Proximal tubule, distal tubule, and peritubular capillary endothelial cell ultrastructure morphology is shown in Fig. 6. The 8 mmHg pressure group had better proximal and distal tubule ultrastructure morphology that showed intact cell membranes with clear cell boundaries, and intact brush borders compared to the 12 mmHg group. The 12 mmHg group showed swollen nuclei, a tenuous cell membrane, a distant boundary between cells, many vacuolizations, and the brush border was detached from the cell body. This indicates greater injury than in the 8 mmHg group. Vacuolization was not seen as much in the distal tubule of the 8 mmHg group as it was in the 12 mmHg group. The peritubular capillary in the 8 mmHg group showed an intact endothelial cell nucleus, endothelial layer, and basement membrane. Comparatively, the 12 mmHg group showed a swollen endothelial cell nucleus, an edematous endothelial layer, and basement membrane disruption in the peritubular capillary.

Follow up appointments were conducted with all patients within 1 year after laparoscopic nephrectomy. The 1 year follow up levels of BUN were not significantly different between both pressure groups (Table 3). In the 8 mmHg group, one patient had a period of bloody urine after surgery, and one patient had minor complaints of surgical site discomfort during activity. In the 12 mmHg group, two patients had minor complaints of surgical site discomfort during activity. The remaining 40 patients had no complaints.

## Discussion

The increased intraabdominal pressure causes mechanical compression of the inferior vena cava, renal vasculature, and parenchyma [13, 14]. It stimulates sympathetic activity, which is regulated through CO_2_-mediated baroreceptors, and can lead to renal cortical vasoconstriction [15–17]. Venous congestion and decreased renal blood flow leads to tissue hypoperfusion that triggers an inflammatory response [18]. Research on animals showed the pneumoperitoneum pressure of 12–18 mmHg resulted in hypoperfusion that induced the release of inflammatory cytokines, neutrophil migration, and renal cell apoptosis in the outer medulla and cortex [7, 15]. In humans, increased intra-abdominal pressure caused the abdominal or splanchnic regions hypoperfusion with or without hypotension [13, 17], and even a slight increased pressure of 10 mmHg has shown to affect the kidney [3, 17]. While several studies have demonstrated the negative effects of positive-pressure pneumoperitoneum, many institutions still continue to use standard pressure pneumoperitoneum at 12–14 mmHg due to its surgical space convenience. Adverse consequences are not expected during most elective laparoscopic operations in healthy or low-risk individuals, however, it has a significant clinical impact on high-risk patients including the elderly population, cardiac dysfunction patients or critically ill patients [6, 19].

Our study results consistent with the previous study results indicating that CI, SVI, MAP, and end-tidal CO_2_ levels were not significantly different between the low and standard pressure groups [20–22]. In our study, the heart rate in the low pressure group was significantly lower than the standard pressure group. This difference has not been reported in previous studies [20, 22]. One effect of low pressure pneumoperitoneum was reduced postoperative pain may have been due to lower visceral pain secondary to peritoneal stretch receptors [20]. However, we excluded pain effects from the outcome since all subjects received intravenous fentanyl maintenance and QL block during surgery and postoperative pain management. Additionally, normal end-tidal CO_2_ value, level of BIS and TOF were maintained at comparable levels during surgery in both groups to exclude hypercarbia. We hypothesized the higher heart rate in the standard pressure group was a response to the higher inflammatory response due to higher pneumoperitoneum pressure.

During pneumoperitoneum insufflation, we observed an increase in RI ​​indicates the increased intra-abdominal pressure caused a decrease in interlobar arterial blood flow [23]. The renal perfusion is affected by the blood flow and pressure on blood vessels [24]. The impaired blood flow changes the normal blood flow from laminar into turbulent or oscillatory flow that causes the shear stress. The shear stress will stimulate the proinflammatory response which is present on the surface of endothelial cells, increase the expression of endothelial adhesion molecules and their interactions with neutrophils, monocytes that triggers the release of IL-6 [23–27]. Our study showed a higher release of IL-6 during pneumoperitoneum insufflation in the standard pressure group than in the low pressure group. Although CO_2_ and surgical techniques can contribute to the release of proinflammatory cytokines [28], our study showed that an acute, slight increase in intraabdominal pressure results in significantly increased IL-6 levels. Furthermore, using a low pressure pneumoperitoneum could attenuate this response.

Studies on the impact of low versus standard pressure pneumoperitoneum have shown various results. A laparoscopic cholecystectomy study performed with low and standard pressures showed no differences in the increase of IL-6 [29]. Our study results mirrored another laparoscopy study that found significantly higher IL-6 levels in the standard-pressure pneumoperitoneum group than in the low pressure pneumoperitoneum group [16]. Yap et al. validated the previously published study demonstrating the animal models of AKI after nephrectomy resulted in the increased IL-6 [30]. Another animal study showed extrarenal IL-6 production from the liver after unilateral nephrectomy that suggested the elevated cytokine content may be due to the increasing endogenous production [31]. As urine output and serum creatinine were within the normal limit throughout the study, our results suggested that the increased plasma IL-6 level was due to increased endogenous production and not because of decreased renal excretion.

As hypothesized, we found the increasing plasma syndecan-1 corresponded to elevated plasma IL-6 that causes syndecan-1 activation and shedding from the endothelial surface of blood vessels into the bloodstream [30, 32]. In accordance with the degree of inflammation that occurs, the shedding of syndecan-1 increased in both levels of pneumoperitoneum pressure compared to the baseline conditions. However, the plasma syndecan-1 level was lower and syndecan-1 expression on proximal tubular cell was higher in the low pressure group than in the standard pressure group. The duration of laparoscopic nephrectomy is longer than open nephrectomy leads to longer and more profound warm ischemia, and the addition of high pressure pneumoperitoneum use contributes to the syndecan-1 shedding [33]. In early renal injury due to inflammation or ischemia/reperfusion or in kidney transplantation, tubular epithelial cells increase syndecan-1 expression and its shedding into the blood as an adaptive response to repair injured cells and promote cell survival [10, 32, 34, 35]. In further injury, epithelial cells will increasingly lose syndecan-1 and the sustained elevating plasma syndecan-1 with low syndecan-1 expression correlate with the degree of kidney tubular function loss [36, 37].

Our study result showed syndecan-1 to be expressed in proximal and distal tubular epithelial cells, with negative syndecan-1 expression within the glomerular or peritubular vasculature. This result was similar to the study of Adepu and colleagues, who found syndecan-1 in the basolateral layer in proximal tubular epithelial cells in human kidney biopsy samples and hypothesized that the increase in plasma syndecan-1 levels was partly derived from an extravascular source such as the renal tubular epithelial cells [34]. Our findings showed contradictory results to a previous animal study that showed the presence of syndecan-1 protein in the glomerulus and peritubular capillaries [37]. Syndecan-1 may not have been detected in the glomerular endothelium because the dominant proteoglycan expression in glomerular endothelial cells are syndecan-4 [38].

We found the level of plasma sVEGFR-2 was significantly higher when standard pressure was used, while the low pressure pneumoperitoneum attenuated the inflammatory response and produced lower plasma sVEGFR-2 levels. From our observation, tubular epithelial cell VEGFR-2 expression was significantly higher in the standard than in the low pressure group. The increase in tubular epithelial cell VEGFR-2 expression suggested that inflammatory responses occurring in circulation reached the extracellular matrix and renal tubules. The low pressure group produced less inflammation to the kidney with less stimulation of VEGFR-2 in the renal endothelial and tubular epithelial cells compared to a higher synthesis and activation of VEGFR-2 in the standard pressure. As a comparison, a previous study showed that overstimulation of VEGFR-2 induced endothelial proliferation, abnormal angiogenesis, extracellular matrix deposition, and acute tubulointerstitial injury in experimental animals [39, 40]. There is an increase in VGEF-mRNA expression at laparoscopic sites as a response to the injured tissue [40–42]. When inflammation occurs, IL-6 and activated syndecan-1 stimulate the synthesis of VEGF-A molecules and its binding to its regulator VEGFR-2 on the glomerular and peritubular capillaries endothelial cells, as well as tubular epithelial cells [39, 40]. Increasing plasma sVEGFR-2 level and VEGFR-2 expression on tubular epithelial cells to the adjacent endothelial cells depends on the extent of inflammation that results in an increased endothelial permeability [37, 41–43].

The electron microscopy examination showed the low pressure group had intact tubular cell membranes with clear cell boundaries and attached brush borders. These morphologies were healthier when compared to the standard pressure group, which showed greater injury, tenuous tubular cell membranes, brush borders detached from the cell body, and more vacuolizations. The extracellular matrix peritubular endothelial cell was more edematous in the standard pressure pneumoperitoneum group. These results support the application of low pressure pneumoperitoneum resulting in a lower degree of ischemia, less tissue inflammation, that reduced endothelial and tubular epithelial cell injury. Animals treated with various increased pneumoperitoneum pressure gradients showed the loss of tubular epithelial cells and cell apoptosis [17]. In humans, acute tubular necrosis was observed in 44–45% of both open and laparoscopic nephrectomy patients, and 54% of renal biopsy specimens taken from laparoscopic nephrectomy showed subcapsular cortical injury. These injuries indicate that pneumoperitoneum and surgical manipulation causes acute tubular necrosis accompanied by peritubular capillary congestion [42].

**Fig. 7:**
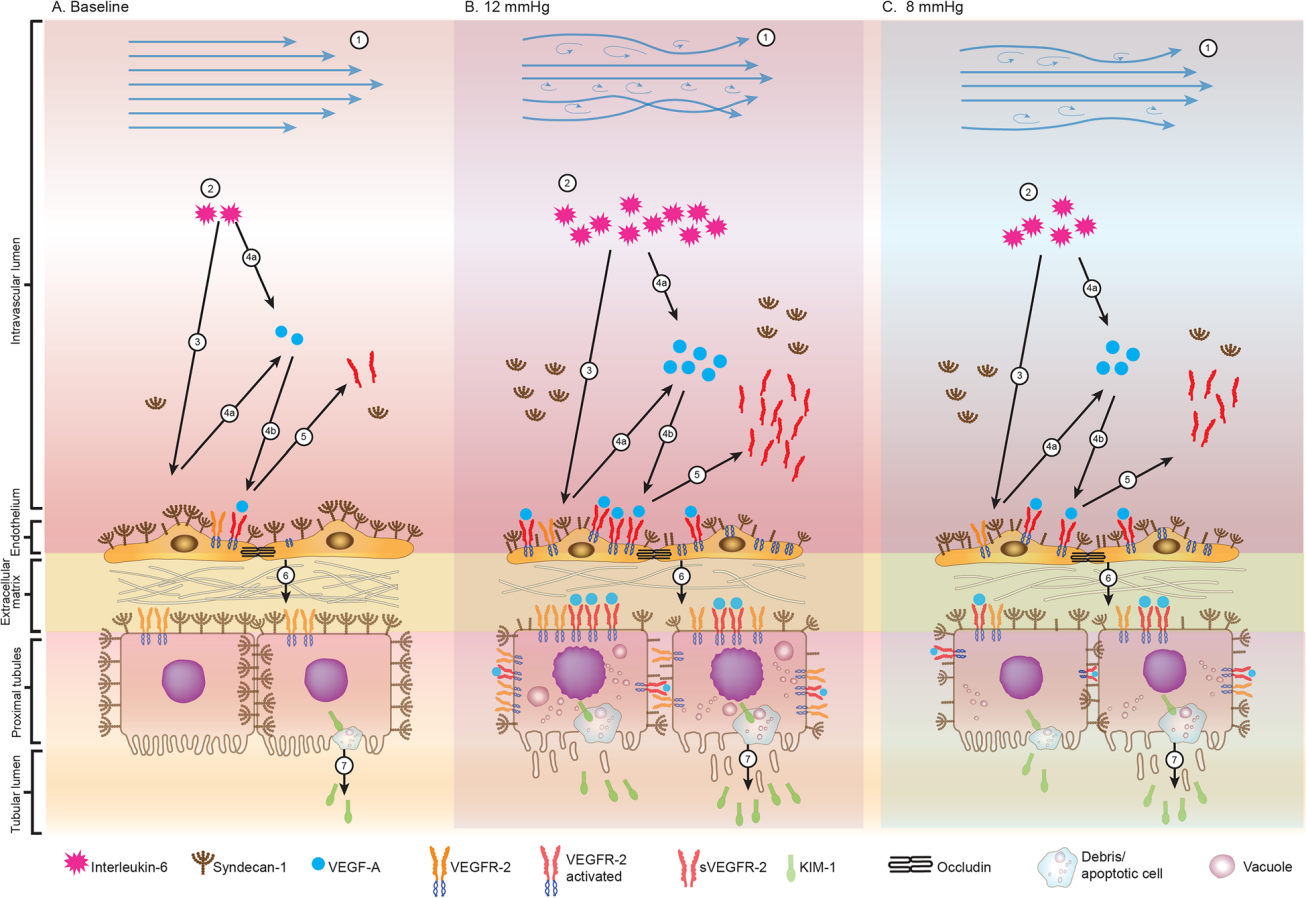
The proposed mechanism of endothelial cell and kidney tubule injury that occurs in the standard and low pressure pneumoperitoneum. Normal baseline condition [**A**]. 1. Standard pressure (12 mmHg group) decreases interlobar artery blood flow and results in more changes from laminar flow to turbulent flow [**B**] than low pressure (8 mmHg group) [**C**]. 2. The inflammatory response in the 12 mmHg group produces higher IL-6 levels than the 8 mmHg group. 3. Interleukin-6 causes more syndecan-1 activation and shedding from the endothelial surface into the bloodstream in the 12 mmHg group than in the 8 mmHg group. 4. (a) Interleukin-6 and syndecan-1 stimulate VEGF-A synthesis and (b) binding to VEGFR-2 on the endothelial surface. 5. Activation of VEGFR-2 increases sVEGFR-2 levels more so in the 12 mmHg group than in the 8 mmHg group. 6. The expression of VEGFR-2 in tubular epithelial cells is higher in the 12 mmHg group, and the expression of syndecan-1 is lower in the 12 mmHg group than in the 8 mmHg group. 7. Due to inflammation, tubular epithelial cell injury stimulates the synthesis of KIM-1 molecules that will be released into the tubular lumen (urine). Figure courtesy of Dita Aditianingsih, MD, PhD. Permission to reuse the figure in any form must be obtained directly from Dr. Aditianingsih

Although the duration of pneumoperitoneum was relatively short, this study results showed the presence of endothelial and renal tubular markers to the inflammation were higher, especially when standard or high pressure pneumoperitoneum was used. The use of low pressure pneumoperitoneum may have attenuated these systemic and vascular inflammatory responses (Fig. 7). IL-6 as an extrarenal mediator of the injury is a clinically important finding. Plasma syndecan-1 level is hypothesized to correlate with plasma soluble VEGF-A and its receptor VEGFR-2, which can be a sensitive marker to detect endothelial and epithelial injury due to perfusion disturbance and inflammation [34, 35]. Both increasing plasma syndecan-1 and sVEGFR-2 levels, rather than plasma creatinine, BUN or urine output, has been proposed as an early marker of the underlying AKI [37]. We observed that the urinary KIM-1 level was not significantly different between low pressure and standard pressure pneumoperitoneum. The KIM-1 level returned to baseline levels 2 h after desufflation represented the reversible tubular injury [44], that may be due to the short length of the pneumoperitoneum duration during laparoscopy procedure. Reducing the syndecan-1 shedding and the release of VEGFR-2 are believed to have renal-protective roles [32, 35, 37, 39]. However, the inhibition of syndecan-1 shedding and sVEGFR-2 response to endothelial injury in preventing or reducing kidney injury demands further experimental and clinical studies.

The limitation of this study was the use of a standard CO2 pressure insufflator which did not allow to keep the intraabdominal pressure always stable during the procedure, especially during suctioning or insertion of laparoscopic instruments that was overcome by intermittent increase in CO2 insufflation pressure. It is also important to evaluate the risks and benefits between low and standard pressure in operator’s point of view related to operative comfort such as space for dissection, and vision while using suction. From the 1 year follow up, all the observed differences are not clinically significant in the long term because of the study is underpowered to detect those differences.

## Conclusion

Our findings demonstrate that using a low pressure pneumoperitoneum attenuated the inflammatory response, measured by quantifying plasma IL-6. This may have caused the observed reductions in syndecan-1 shedding and VEGFR-2 expression; the renal tubular and vascular endothelial proinflammatory markers of injury in response to the presence of systemic inflammatory cytokines. Therefore, we should consider using lower pneumoperitoneum pressure during laparoscopic nephrectomy. Lowering pneumoperitoneum pressure is a logical modification to implicate in an effort to reduce endothelium and renal tubular epithelium injury.

## Supplementary information

**Additional file 1.** Quadratus Lumborum Block.

**Additional file 2.** The port placement and surgical space condition during 12 mmHg pressure pneumoperitoneum.

**Additional file 3.** The port placement and surgical space condition during 8 mmHg pressure pneumoperitoneum.

**Additional file 4.** Comparison of renal resistive index (RI), plasma interleukin-6 (IL-6), syndecan-1, soluble VEGFR-2, urinary KIM-1, syndecan-1 and tubular epithelial VEGFR-2 expression between 12 mmHg and 8 mmHg groups.

## Data Availability

The datasets used and/or analyzed in the current study are available from the corresponding author upon reasonable request.

## Abbreviations

AKI: Acute kidney injury
BM: Basement membrane
IAP: Intra-abdominal pressure
IL-6: Interleukin-6
KIM-1: Kidney injury molecule-1
QL: Quadratus lumborum
RI: Resistive index
sVEGFR-2: Soluble vascular endothelial growth factor receptor-2
VEGF: Vascular endothelial growth factor
