# Supplementary material for: Effects of low versus standard pressure pneumoperitoneum on renal syndecan-1 shedding and VEGF receptor-2 expression in living-donor nephrectomy: a randomized controlled study
Source: BMC Anesthesiol. 2020 Feb 4;20:37. doi: 10.1186/s12871-020-0956-7 (PMC7001365; doi:10.1186/s12871-020-0956-7)
Supplement: Supplementary file 1 — Additional file 1. Quadratus Lumborum Block. [file 12871_2020_956_MOESM1_ESM.docx]

**Quadratus Lumborum Block Method Description**

Patients were in the supine position with the site to be blocked slightly facing upward. This position was facilitated by a pillow underneath the patient and table tilting. After ensuring skin asepsis of the area, a 2.0–5.5 MHz convex transducer (4C-RS, Logic e, GE Healthcare U.S.A; C5-1E, DC-70, Mindray, Shenzen, China) covered with sterile drapes was attached to the inferior area of the lumbar (Petit's triangle that consisted of the iliac crest in the inferior region, the latissimus dorsi muscle in the posterior region, and the external abdominal oblique muscle in the anterior region). The Shamrock sign appeared on the ultrasound, and a 21G 100-mm peripheral block needle (Stimuplex^®^, BBraun, Mesulngen, Germany) was inserted in-plane with the USG probe passing in an anterior to posterior direction through the quadratus lumborum (QL) muscle and reaching the border between the QL and psoas major muscle. After confirming negative blood aspiration, 1 mL normal saline was injected to obtain a hydrodissection sign to verify the needle tip, and 0.4 mL/kg of bupivacaine 0.25% with a maximum of 20 mL was injected on each side.
