# Supplementary material for: Effects of low versus standard pressure pneumoperitoneum on renal syndecan-1 shedding and VEGF receptor-2 expression in living-donor nephrectomy: a randomized controlled study
Source: BMC Anesthesiol. 2020 Feb 4;20:37. doi: 10.1186/s12871-020-0956-7 (PMC7001365; doi:10.1186/s12871-020-0956-7)
Supplement: Supplementary file 4 — Additional file 4. Comparison of renal resistive index (RI), plasma interleukin-6 (IL-6), syndecan-1, soluble VEGFR-2, urinary KIM-1, syndecan-1 and tubular epithelial VEGFR-2 expression between 12 mmHg and 8 mmHg groups. [file 12871_2020_956_MOESM4_ESM.docx]

**Figure S2.** Comparison of renal resistive index (RI), plasma interleukin-6 (IL-6), syndecan-1, soluble VEGFR-2, and urinary KIM-1 between 12 mmHg and 8 mmHg groups.

| **Parameters** | **12 mmHg** | ***p*-value*** | **8 mmHg** | ***p*-value*** | ***p*-value**** | **Mean difference (95% CI)** | ***p*-value***** |
| --- | --- | --- | --- | --- | --- | --- | --- |
| **Renal resistive index** |  |  |  |  |  |  |  |
| a. Baseline | 0.59 (0.55–0.62) |  | 0.60 (0.55–0.61) |  | 0.9 | -0.071 (-0.105– -0.037) |  |
| b. 2 hours of pneumoperitoneum | 0.66 (0.63–0.68) | <0.001 | 0.67 (0.65–0.70) | <0.001 | 0.4 | 0.0185 (0.009–0.654) | 0.8 |
| c. 2 hours after desufflation | 0.66 (0.64–0.68) |  | 0.68 (0.66–0.70) |  | 0.4 | 0.015 (0.012–0.02) |  |
| **Interleukin-6 (pg/mL)** |  |  |  |  |  |  |  |
| a. Baseline | 1.66 (1.41–1.90) |  | 1.50 (1.31–1.69) |  | 0.3 | 1.10 (1.07–1.12) |  |
| b. 2 hours of pneumoperitoneum | 8.92 (6.21–11.62) | <0.001 | 4.75 (3.50–5.99) | <0.001 | 0.003 | 1.86 (1.78–1.94) | 0.1 |
| c. 2 hours after desufflation | 46.17 (35.36–56.98) |  | 37.42 (27.89–46.95) |  | 0.2 | 1.24 (1.21–1.27) |  |
| **Syndecan-1 (ng/mL)** |  |  |  |  |  |  |  |
| a. Baseline | 10.87 (8.81–12.92) |  | 12.07 (9.56–14.57) |  | 0.5 | 0.91 (0.69–1.20) |  |
| b. 2 hours of pneumoperitoneum | 15.18 (11.14–19.22) | <0.001 | 13.66 (10.04–17.27) | <0.001 | 0.1 | 1.34 (0.94–0.92) | 0.1 |
| c. 2 hours after desufflation | 30.52 (23.80–37.23) |  | 33.12 (25.21–41.02) |  | 0.9 | 0.93 (0.91–0.94) |  |
| **sVEGFR-2 (pg/mL)** |  |  |  |  |  |  |  |
| a. Baseline | 7836.97 (7052.84–8621.09) |  | 7635.32 (6619.80–8650.83) |  | 0.8 | 196.48 (-1046.08–1439.05) |  |
| b. 2 hours of pneumoperitoneum | 8106.02 (7187.38–9024.66) | 0.1 | 6841.05 (5598.85–8083.25) | 0.4 | 0.032 | 1264.93 (941.41–1588.53) | 0.1 |
| c. 2 hours after desufflation | 8452.25 (7486.88–9417.61) |  | 7263.92 (6258.32–8269.51) |  | 0.044 | 1188.33 (1148.10–1228.57) |  |
| **KIM-1 (ng/mL)** |  |  |  |  |  |  |  |
| a. Baseline | 0.32 (0.18–0.45) |  | 0.52 (0.36–0.68) |  | 0.043 | 0.54 (0.44–0.67) |  |
| b. 2 hours of pneumoperitoneum | 0.47 (0.33–0.60) | <0.001 | 0.51 (0.38–0.64) | <0.001 | 0.7 | 0.93 (0.92–0.94) | 0.2 |
| c. 2 hours after desufflation | 0.20 (0.12–0.27) |  | 0.21 (0.15–0.27) |  | 0.7 | 0.92 (0.83–1.03) |  |

Data presented in geometric mean and confidence interval 95% (minimum–maximum); *repeated ANOVA test, *p* < 0.05 is significant; **unpaired t-test, *p* < 0.05 is significant; ***general linear model test, *p* < 0.05 is significant

**Figure S3.** Syndecan-1 expression of tubular epithelial cells in 12 mmHg and 8 mmHg groups.

| **Syndecan-1 H-score** | **12 mmHg** | **8 mmHg** | ***p*-value*** |
| --- | --- | --- | --- |
| Proximal tubule | 211.00 (199.05–219.67) | 225.90 (215.46–231.50) | 0.03 |
| Distal tubule | 108.10 (98.49–118.31) | 112.80 (94.53–128.12) | 0.8 |

Data are presented as median (95% CI). The two groups were compared using Mann-Whitney test; * *p* < 0.05 is significant.

**Figure 4.** Tubular epithelial VEGFR-2 expression in the 12 mmHg and 8 mmHg groups

| **SVEGFR-2 H-score** | **12 mmHg** | **8 mmHg** | ***p*-value*** |
| --- | --- | --- | --- |
| Proximal tubule | 278.00 (269.37–282.05) | 258.80 (248.93–268.91) | 0.005 |
| Distal tubule | 288.80 (282.59–291.37) | 279.40 (271.36–284.72) | 0.02 |

Data are presented as median (95% CI). The two groups were compared using Mann-Whitney test; * *p* < 0.05 is significant.
